# Glycosaminoglycan from *Apostichopus japonicus* Improves Glucose Metabolism in the Liver of Insulin Resistant Mice

**DOI:** 10.3390/md18010001

**Published:** 2019-12-18

**Authors:** Yunmei Chen, Yuanhong Wang, Shuang Yang, Mingming Yu, Tingfu Jiang, Zhihua Lv

**Affiliations:** 1 Key Laboratory of Marine Drugs, Ministry of Education of China, Key Laboratory of Glycoscience & Glycotechnology of Shandong Province, School of Medicine and Pharmacy, Ocean University of China, Qingdao 266003, China; yunmchen@hotmail.com (Y.C.); yhwang@ouc.edu.cn (Y.W.); yangshuang@ouc.edu.cn (S.Y.); jiangtingfu@ouc.edu.cn (T.J.); 2 Laboratory for Marine Drugs and Bioproducts of Qingdao National Laboratory for Marine Science and Technology, Qingdao 266003, China

**Keywords:** glycosaminoglycan, Apo*stichopus japonicus*, glucose metabolism, Akt, AMPK

## Abstract

Holothurian glycosaminoglycan isolated from *Apostichopus japonicus* (named AHG) can suppress hepatic glucose production in insulin resistant hepatocytes, but its effects on glucose metabolism in vivo are unknown. The present study was conducted to investigate the effects of AHG on hyperglycemia in the liver of insulin resistant mice induced by a high-fat diet (HFD) for 12 weeks. The results demonstrated that AHG supplementation apparently reduced body weight, blood glucose level, and serum insulin content in a dose-dependent manner in HFD-fed mice. The protein levels and gene expression of gluconeogenesis rate-limiting enzymes G6Pase and PEPCK were remarkedly suppressed in the insulin resistant liver. In addition, although the total expression of IRS1, Akt, and AMPK in the insulin resistant liver was not affected by AHG supplementation, the phosphorylation of IRS1, Akt, and AMPK were clearly elevated by AHG treatment. These results suggest that AHG could be a promising natural marine product for the development of an antihyperglycemic agent.

## 1. Introduction

Type 2 diabetes mellitus(T2DM) is characterized by long-term persistent hyperglycemia with several complications such as eye injury, renal failure, cardiovascular disease, and nervous system damage [1]. According to a report from the International Diabetes Federation (IDF) published in 2017, the number of people with diabetes has risen to 425 million in the world (2017, IDF, http://diabetesatlas.org) and T2DM makes up about 90% of cases of diabetes [2]. Thus, T2DM has become a chronic disease globally. Insulin resistance is the hallmark of T2DM and describes a condition whereby the ability of insulin to trigger glucose uptake, metabolism, or storage is impaired [3,4]. As the major site of glucose utilization during the post-prandial period and the main human tissue of glucose synthetization, the liver is the vital target organ of insulin resistance, and liver insulin resistance is the main reason for the development of T2DM [5,6]. When insulin resistance develops in the liver, increased hepatic glucose production and decreased glucose utilization contribute to the elevated level of blood glucose [7]. Therefore, an increasing number of studies are examining the liver, and specifically the inappropriate glucose metabolism in insulin resistant individuals.

Up to now, due to the huge species biodiversity and extremely harsh living environment, marine organisms have been found to have biologically active agents which show antidiabetic effects, and the sea cucumber is one of the most widely used marine traditional medicines in Asia [8,9]. Sea cucumbers are echinoderms from the class Holothuroidea with a wide spectrum of biological and pharmacological activities, such as anti-cancer [10], anticoagulant [11], antifungal [12], anti-hypertension [13], antioxidant [14], anti-inflammatory [15], anti-diabetic [16] and antiviral [17] activities. Recent studies demonstrate that the sea cucumber exerts various activities on account of the high content of bioactive constituents, including saponins, polypeptides, gangliosides, and polysaccharides [18,19]. Polysaccharides from sea cucumber are divided into two groups, namely holothurian fucan and holothurian glycosaminoglycan. Holothurian glycosaminoglycan is a kind of highly sulfated polysaccharide isolated from the sea cucumber body wall, possessing a chondroitin sulfate backbone with a high percentage of sulfated α-L-fucose (Fuc) branches [20]. Among the various biological macromolecules, holothurian glycosaminoglycan is considered as the major bioactive constituent of sea cucumber [21].

**Figure 1:**
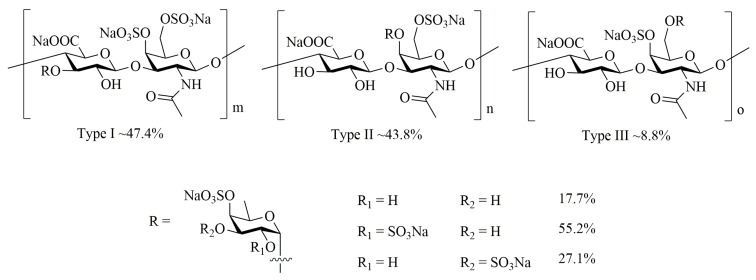
Chemical Structure of AHG from Sea Cucumber *Apostichopus japonicus*.

*Apostichopus japonicus* is an economically important sea cucumber which is widely distributed in Russia, China, Japan and Korea [22]. AHG (the structure shown in Figure 1) is a novel glycosaminoglycan from *Apostichopus japonicus* with a chondroitin sulfate-like backbone structure of →4GlcA(Fuc2S,4Sα1→3)β1→3GalNAc4S6Sβ1→[23]. In our previous study, AHG exhibits anti-diabetic activity by suppressing hepatic glucose production in insulin resistant hepatocytes [24]. However, the physiological effects of AHG in vivo are unknown. In this study, we investigated the protective ability of AHG on dysregulated glucose homeostasis in insulin resistant mice induced by a high-fat diet (HFD). Also, we further explored the possible biochemical regulator involving in the effects of AHG in the liver glucose metabolism of HFD-fed insulin resistant mice.

## 2. Results

### 2.1. AHG Decreased Body Weight in Mice Fed with HFD

**Figure 2:**
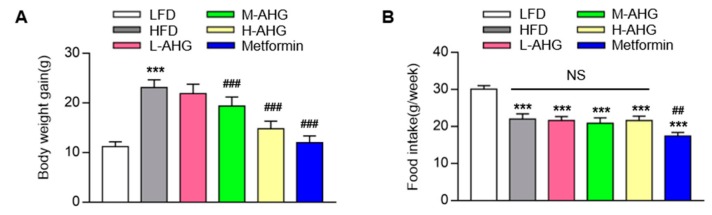
Effects of AHG supplementation on body weight in insulin resistant mice induced with HFD. C57BL/6J mice were fed with HFD for 12 weeks and treated with low, medium and high doses (20, 50, and 100 mg/kg/day, respectively) of AHG for eight weeks. (**A**) Body weight gain; (**B**) food intake per week. Data are showed as mean ± SD, *n* = 8; *p < 0.5, **p < 0.1, ***p < 0.01 vs. LFD group, ^#^*p* < 0.5, ^##^*p* < 0.1, ^###^*p* < 0.01 vs. HFD group.

As shown in Figure 2A, after 12 weeks of HFD treatment, mice exerted two-fold increase in body weight gain compared to mice of LFD group. AHG (20 mg/kg/day) supplementation showed no significant change in body weight gain compared to HFD group, however, treatment of AHG (50 mg/kg/day) and AHG (100 mg/kg/day) alleviated the enhancement by showing 12.1% and 18.9% inhibition in the body weight gain of the HFD group, indicating that AHG affected an HFD-induced insulin resistant model. As a positive control, the body weight gain of mice fed metformin was 24% lower than that of mice fed HFD. To further detect whether the decreasing body weight was caused by the change of energy intake, the food intake was measured. The results showed (Figure 2B) that food intake was significantly lower in the HFD group compared to the LFD group. However, AHG supplementation showed no effect on food intake in HFD treatment. Metformin showed a markedly suppression on food intake compared to LFD group, which was consistently in agreement with the literature [25].

### 2.2. AHG Improved Glucose Metabolism in Mice Fed with HFD

**Figure 3:**
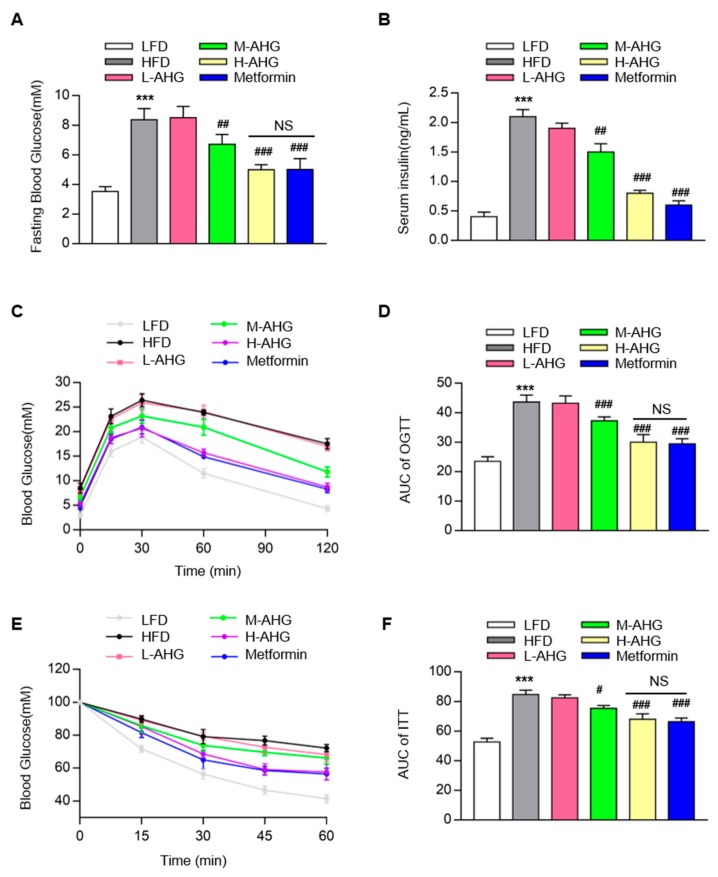
Effects of AHG supplementation on glucose metabolism in insulin resistant mice induced with HFD. C57BL/6J mice were fed with HFD for 12 weeks and treated with low, medium and high doses (20, 50 and 100 mg/kg/day, respectively) of AHG for eight weeks. (**A**) Fasting blood glucose; (**B**) serum insulin content; (**C**) Oral glucose tolerance test (OGTT); (**D**) The values of AUC for OGTT; (**E**) Insulin tolerance test (ITT); (**F**) The values of AUC for ITT. Data are showed as mean ± SD, *n* = 8; *p < 0.5, **p < 0.1, ***p < 0.01 vs. LFD group, ^#^*p* < 0.5, ^##^*p* < 0.1, ^###^*p* < 0.01 vs. HFD group.

As shown in Figure 3A, compared to LFD group, an obvious increase in fasting blood glucose levels was observed in HFD group (*p* < 0.01), however, the increasement was attenuated by AHG in a dose-dependent manner. Also, fasting plasma glucose in H-AHG group was similar to Metformin group, indicating that AHG supplementation (100 mg/kg/day) had a similar hypoglycemic effect as metformin. HFD impaired glucose tolerance sharply, which was attenuated by the supplementation of AHG in a concentration-dependent pattern (Figure 3C–D). Consistent with this result, insulin injection failed to decline blood glucose in HFD mice, whereas blood glucose decreased normally in H-AHG mice compared to HFD mice, which was reflected in the area under the curve for ITT (Figure 3E–F). Moreover, no significant difference of blood glucose level was found between H-AHG group and Metformin group in OGTT (*p* = 0.48) and ITT (*p* = 0.25) (Figure 3C–E). The consumption of the HFD mice also caused high level of serum insulin, which was increased four-fold when compared with the basal level of insulin content in LFD mice (Figure 3B). However, this effect was abolished in the H-AHG group. There was no notable difference in the serum insulin content between the H-AHG and Metformin groups. Overall results confirmed that the treatment of AHG improved insulin resistance induced by HFD in C57BL/6J mice.

### 2.3. AHG alleviated liver injury in mice fed with HFD

**Figure 4:**
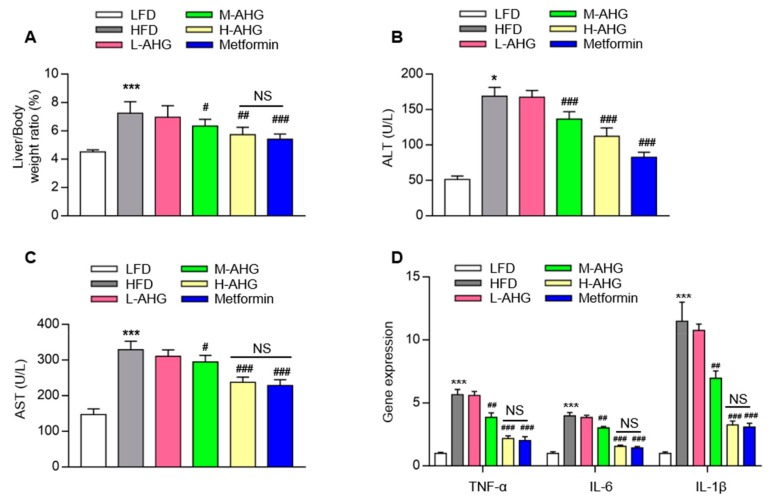
Effects of AHG supplementation on liver injury and inflammation induced by HFD. C57BL/6J mice were fed with HFD for 12 weeks and treated with low, medium and high doses (20, 50 and 100 mg/kg/day, respectively) of AHG for eight weeks. (**A**) Liver/body weight ratio; (**B**) The values of ALT; (**C**) The values of AST; (**D**) The mRNA expression analysis of TNF-α, IL-6 and IL-1β were measured by RT-PCR and normalized by Cyclophilin. Data are showed as mean ± SD, *n* = 8; *p < 0.5, **p < 0.1, ***p < 0.01 vs. LFD group, ^#^*p* < 0.5, ^##^*p* < 0.1, ^###^*p* < 0.01 vs. HFD group

Considering that the liver is the main target tissue of insulin resistance, we next explored whether AHG affected liver tissue in HFD-induced insulin resistance mice. The liver tissue weight, ALT level and AST level were measured. As shown in Figure 4A, a high dose of AHG significantly decreased the liver/body weight ratio in HFD mice (*p* < 0.001). Additionally, the value of ALT and AST showed the similar trend, indicating that AHG alleviated liver injury caused by HFD (Figure 4B,C). Gene expression analysis indicated that HFD stimulated inflammatory cytokines transcriptional levels of TNF-α, IL-6 and IL-1β in control mice by 5.7, 4.0, and 11.5-fold, respectively. The elevation of the gene expression of TNF-α, IL-6 and IL-1β was reduced when mice fed with high dose AHG (Figure 4D). These results indicate that AHG attenuated spontaneous liver injury and inflammation caused by HFD.

### 2.4. AHG Suppressed Hepatic Gluconeogenesis in Fasting Mice Fed A High Fat Diet

**Figure 5:**
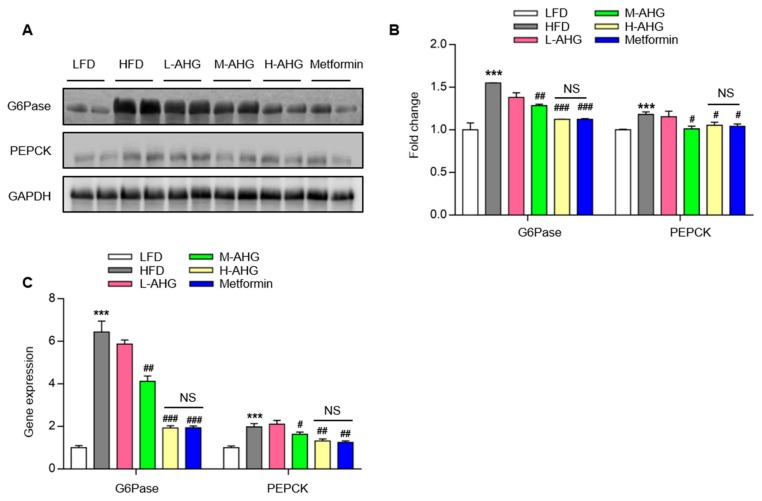
Effects of AHG supplementation on gluconeogenesis in insulin resistant mice induced with HFD. C57BL/6J mice were fed with HFD for 12 weeks and treated with low, medium and high doses (20, 50 and 100 mg/kg/day, respectively) of AHG for eightweeks. (**A**) The protein levels of G6Pase and PEPCK in livers were measured by Western blot. (**B**) Quantification of protein levels of G6Pase and PEPCK was performed by Image J. GAPDH as a control to normalize the expression of the protein. Data are showed as mean ± SD, *n* = 3; (**C**) Gene expression of G6Pase and PEPCK in livers were quantified by RT-PCR. Data are shown as mean ± SD, *n* = 4; *p < 0.5, **p < 0.1, ***p < 0.01 vs. LFD group, ^#^*p* < 0.5, ^##^*p* < 0.1, ^###^*p* < 0.01 vs. HFD group.

Our previous study showed that AHG suppressed hepatic gluconeogenesis in insulin resistant hepatocytes [24]. Next, we determined whether AHG affected hepatic gluconeogenesis in an insulin resistant liver. As shown in Figure 5, the protein level and gene expression of gluconeogenesis rate-limiting enzymes G6Pase and PEPCK were measured. In the livers, HFD significantly increased the protein level of G6Pase and PEPCK by 50% and 20%, respectively, but the increased potential in mice with the administration of high dose AHG was, respectively, 12% and 5%, with a significant reduction compared to HFD mice (Figure 5A,B). The Q-PCR result showed the same trend. As a positive control, metformin repressed the transcriptional and translational level of G6Pase and PEPCK as reported in the literature [26]. This confirms that AHG suppressed hepatic gluconeogenesis in insulin resistant mice fed with a HFD.

### 2.5. AHG Improved Insulin Signaling Pathway in Liver of Mice Fed with HFD

**Figure 6:**
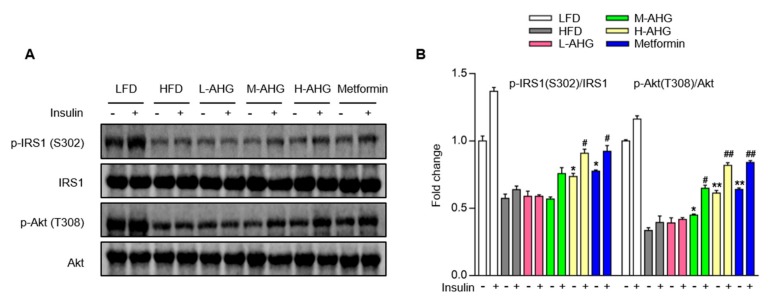
Effects of AHG supplementation on insulin signaling pathway in the livers of insulin resistant mice induced with HFD. C57BL/6J mice were fed with HFD for 12 weeks and treated with low, medium and high doses (20, 50 and 100 mg/kg/day, respectively) of AHG for eight weeks. At the end of treatment, mice were fasted for 12 h and intraperitoneally injected with either saline or insulin (20 units/kg). (**A**) The protein expression of p-IRS (S302), IRS1, p-Akt (T308) and Akt were measured by Western blot. (**B**) Quantification of p-IRS (S302)/IRS1 and p-Akt (T308)/Akt was performed by Image J. Data are showed as mean ± SD, *n*=3; *p < 0.5, **p < 0.1, ***p < 0.01 vs. insulin untreated HFD group, ^#^*p* < 0.5, ^##^*p* < 0.1, ^###^*p* < 0.01 vs. insulin treated HFD group.

To explore the effect of AHG on insulin signaling pathway in liver, mice were administered with or without insulin by intraperitoneal injection 2 min before euthanasia. The protein level of major cascades of insulin signaling pathway were assessed by Western Blot. HFD consumption suppressed the basal and insulin-stimulated protein level of p-IRS1(S302) and p-Akt (T308), supplementation of AHG resulted in a dose-dependent increase of IRS1 and Akt protein level in basal and insulin-stimulated state (Figure 6A,B). Metformin also activated the insulin signaling cascade pathway, which was consistent with the previously reported work [27]. The above results suggest that the observed alterations in glucose metabolism caused by HFD are associated with an impaired IRS1/Akt pathway in liver, and this effect is mitigated by AHG supplementation.

### 2.6. AHG Activated AMPK in Liver of Mice Fed a High Fat Diet

**Figure 7:**
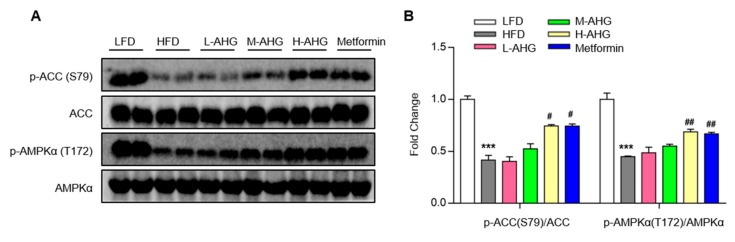
Effects of AHG supplementation on AMPK signaling pathway in the livers of insulin resistant mice induced with HFD. C57BL/6J mice were fed with HFD for 12 weeks and treated with low, medium and high doses (20, 50 and 100 mg/kg/day, respectively) of AHG for eight weeks. (**A**) The protein expression of p-ACC (S79), ACC, p-AMPKα (T172) and AMPKα were measured by Western blot. (**B**) Quantification of p-ACC (S79)/ACC and p-AMPKα (T172)/ AMPKαwas performed by Image J. Data are showed as mean ± SD, *n* = 3; * *p* < 0.5, **p < 0.1, ***p < 0.01 vs. LFD group, ^#^ *p* < 0.5, ^##^ *p* < 0.1, ^###^ *p* < 0.01 vs. HFD group.

AMPK and Akt are the two primary effectors in response to glucose metabolism [28]. AMPK maintains blood glucose levels and reduces hepatic gluconeogenesis gene expression-PEPCK and G6Pase. It is currently unknown that whether AHG activates AMPK in the insulin resistant liver. To address whether AMPK was involved in the effect of AHG on glucose homeostasis in liver, the protein levels of phosphorylated AMPK and an AMPK substrate, acetyl-CoA carboxylase (ACC) were measured. As illustrated in Figure 7, in the liver of insulin resistance mice induced by HFD, AHG elevated the p-AMPK and p-ACC in a dose-dependent manner. Also, there was no difference in activating AMPK between H-AHG and Metformin groups. These results state clearly that the possible role of AHG on repressing hepatic gluconeogenesis via AMPK signaling pathway.

## 3. Discussion

Having high nutritional and medicinal qualities, sea cucumber has been drawn notable attention [9]. There is culminating evidence that polysaccharides from sea cucumber have an effective anti-diabetic activity [16,29,30,31]. In our previous study, AHG was shown to have a therapeutic effect on hepatic glucose metabolism in vitro, however, there is no report target the effect of AHG on glucose metabolism in vivo. In the current study, we provided evidence that AHG treatment significantly alleviated fasting blood glucose and improved insulin resistance by suppressing gluconeogenesis in HFD-fed insulin resistant mice. Also, AHG supplementation elevated IRS1/Akt activation and AMPK phosphorylation in the liver of insulin resistant mice induced by HFD. All of these data suggest that AHG improved glucose metabolism in vivo.

The liver plays a crucial role in organismic energy metabolism, especially glucose metabolism, and dysfunction of the liver can result in acute metabolic abnormalities [32,33]. Due to the primary site of insulin clearance, liver is associated with the development of insulin resistance. In insulin resistance, the increased gluconeogenesis is the main mechanism involving in elevated blood glucose [34]. In the present study, the effect of AHG on gluconeogenesis was detected. HFD supplementation significantly enlarged the gene expression and protein levels of G6Pase and PEPCK in the liver, and that administration with high dose AHG remarkably inhibited this trend. These results implied that AHG maintained glucose metabolism in the liver via suppressing gluconeogenesis.

Insulin is the crucial hormone regulating gluconeogenesis and insulin represses gluconeogenesis by activating the insulin signaling pathway [35]. Insulin binds to insulin receptor and then activates phosphorylation of insulin receptor substrate (p-IRS), followed by recruitment and phosphorylation of Akt. These are key protein kinases involving in restraining gluconeogenesis related genes G6Pase and PEPCK [36,37]. Therefore, the activation of insulin signaling pathway in liver may be a promising strategy for improving insulin sensitivity. In the current study, HFD impaired the phosphorylation of IRS1 and Akt induced by insulin in the liver. However, AHG supplementation alleviated these alterations. Also, AHG improved insulin tolerance, glucose tolerance and alleviated serum insulin content in HFD group mice, suggesting that AHG improved insulin sensitivity induced by HFD, these provided insight into the anti-insulin resistance actions of AHG.

As an intracellular energy sensor, AMPK is another well-known suppressor of gluconeogenesis in addition to insulin pathway and the suppression of AMPK accompanies insulin resistance [38]. Thus, AMPK is considered as a potential target for diabetes prevention and insulin resistance treatment. So far, cumulative research has reported that various natural products regulated glucose metabolism through AMPK, such as epigallocatechin-3-gallate (EGCG) [39], ginsenoside Rg1 [40], rosmarinic acid [41], and astragalus polysaccharide [42]. To the best of our knowledge, it is barely published to target the polysaccharide isolated from marine organism especially sea cucumber on the effect of glucose metabolism through AMPK. Here, we explored how AHG activated AMPK in an insulin resistant liver induced by HFD. Thus, one of the mechanisms of the effect of AHG on glucose homeostasis in insulin resistance mice might be related to AMPK pathway.

Chronic inflammation plays a vital role in the process of liver injury induced by insulin resistance and associated with disease risk [43]. It is reported that proinflammatory cytokines TNF-α and IL-6 produced by HFD were able to induce insulin resistance [44]. In inflammation, proinflammatory cytokine is reported to activate various serine kinases such as c-Jun N-terminal kinase (JNK) [45], S6 kinase (S6K) [46], double-stranded RNA-dependent protein kinase mammalian [47], and inhibitor of nuclear factor kappa-B kinase subunit β (IKKβ) [48]. These serine kinases can alleviate the activation of IRS1, resulting in the impairment of insulin receptor-mediated signaling and the occurrence of insulin resistance. In our HFD-induced insulin resistant model, AHG attenuated HFD-induced liver inflammation by inhibiting the expression of inflammatory genes. The anti-inflammatory effect of AHG on the liver may be one potential explanation for the beneficial effects of AHG on insulin resistance.

The high molecular weight is thought to be the main factor that suppress the transepithelial transport of glycosaminoglycan. It is reported that a fucosyl chondroitin sulfate chains (Mw=12.00 KDa) isolated from sea cucumber can be detected in plasma and urine after oral administration (50 mg/kg) [49]. Additionally, a glycosaminoglycan from Cucumaria frondosa (Mw=21.53 kDa) can improve glucose metabolism in the liver of the insulin resistant mice after oral administration [30]; a glycosaminoglycan from Acaudina molpadioides (Mw=21.53 kDa) was found to improve glucose metabolism in the liver and skeletal muscle of the insulin resistant mice after oral administration [16,29]. A sulfated polysaccharide from sea cucumber Stichopus japonicus (SCSP, Mw=179.4 kDa) can prevent obesity in association with modification of gut microbiota in HFD-fed mice [31]. These studies indicated that glycosaminoglycans from sea cucumber have the possibility to be absorbed in liver and muscle skeletal of insulin resistant mice. Based on our previously study (data unpublished), AHG is a moderate absorption drug in an endocytosis manner using Caco-2 and M cell models. In Caco-2 and M cell models, the Papp (AP-BL) values of AHG were about 2 ×10^-6^ cm/s and 8 ×10^-6^ cm/s, respectively. This indicates AHG is absorbed in the intestinal tracts. The pharmacokinetic study of AHG in animals will be of interest for further study.

Cumulative evidence supports a beneficial effect of AHG on glucose homeostasis as we observed in the model of HFD-induced insulin resistant mice. Oral treatment with AHG significantly reduced body weight and fasting blood glucose level. Furthermore, AHG supplementation improved insulin resistance by repressing gluconeogenesis related genes. In addition, the activation of IRS1/Akt and AMPK signaling pathway are the possible mechanisms underlying the effect of AHG on glucose metabolism in insulin resistant liver. In summary, these findings provide evidence that AHG has the potential to become a novel marine natural product to provide a therapy for the prevention and treatment of insulin resistance and type 2 diabetes. To have a better understanding of the effect of AHG on glucose metabolism in vivo, further investigation is necessary to verify the effect of AHG on regulation of glucose homeostasis in other insulin target tissues, such as skeletal muscle and adipose tissues.

## 4. Materials and Methods

### 4.1. Chemical Reagents

Insulin and metformin were obtained from Aladdin (Shanghai, China). Antibodies PEPCK, G6Pase, p-IRS1(S302), p-Akt (T308), IRS1, Akt, p-AMPKα (T172), p-ACC (S79), AMPKα and ACC were all purchased from Cell Signaling Technology (Danvers, MA, United States). Insulin was obtained from Millipore Sigma (Danvers, MA, United States). Radioimmunoprecipitation assay buffer (RIPA buffer), SDS-PAGE, PVDF membrane and ECL were obtained from Beyotime (Shanghai, China).

### 4.2. Preparation of AHG

AHG was prepared as previously described [23]. Specifically, AHG was extracted from the body wall of the sea cucumber Apostichopus japonicus, purchased from the Nanshan market of Qingdao, China. AHG was prepared as previously described. The body wall of fresh sea cucumber Apostichopus japonicus was grinded into homogenate and extracted with KOH (1 mol/L) at 60 °C for 60 min. After neutralization with cold HCl, diastase vera (EC 3.3.21.4) was added to hydrolyze the protein. The crude polysaccharide was precipitated with 60% ethanol. The crude AHG was further fractionated by a Q Sepharose Fast Flow column (300 mm × 30 mm) with elution by a step-wise gradient of 0.75 and 1.5 M NaCl. The fractions eluted with 1.5 M NaCl were further purified on a Sephadex 25 column (100 × 2.6 cm) with deionized water at a flow rate of 0.3 mL/min. The purified AHG were pooled and lyophilized. The yield of AHG isolated from the fresh sea cucumber Apostichopus japonicus was 0.51% by weight. The average molecular weight of AHG was 98.07 kDa and the purity of AHG was over 99% by using gel filtration chromatography. Monosaccharide composition analysis based on pre-column derivatization reversed-phase HPLC showed that AHG consisted of glucuronic acid, galactosamine and fucose in the molar ratio of 1/1.03/1.16. The sulfate content was 33.20% determined by high performance capillary electrophoresis.

### 4.3. Animals and Animal Care

Male C57BL/6J mice (18-22 g) were obtained from Jinan Pengyue Experimental Animal Breeding Co. Ltd. (License Number: SCXK (lu) 2014-0007) and housed in a controlled condition (25 °C, 50 ± 5 % humidity and 12 h dark-light cycles). All mice were randomly divided into six groups for 12 weeks (8 mice/group): (1) LFD group, given low fat diet (LFD) for 12 weeks; (2) HFD group, given High Fat Diet (HFD) for 12 weeks; (3) L-AHG group, given HFD for 4 weeks, then given HFD simultaneously with oral administration of low dose AHG (20 mg/kg/day) for another 8 weeks; (4) M-AHG group, given HFD for 4 weeks, then given HFD simultaneously with oral administration of medium dose AHG (50 mg/kg/day) for another 8 weeks; (5) H-AHG group, given HFD for 4 weeks, then given HFD simultaneously with high dose oral administration of AHG (100 mg/kg/day) for another 8 weeks; (6) Metformin group, given HFD for 4 weeks, then given HFD simultaneously with oral administration of metformin (200 mg/kg/day) for another 8 weeks. LFD (TP23523) and HFD (60% fat, TP23520) were purchased from Nantong Teluofei Feed Technology Co., Ltd. (Nantong, China). At the end of treatment, mice were fasted for 12 h and intraperitoneally injected with either saline or insulin (20 units/kg). Mice were euthanized 2 min after injection. Blood were collected and centrifuged at 3000 rpm for 15min at 4 °C to obtain serum. Tissues were weighed and flash frozen in liquid nitrogen. The anti-hyperglycemic action of metformin is mainly a consequence of suppressed glucose output owing to inhibition of liver gluconeogenesis [50]. The centre of mechanism of metformin on liver gluconeogenesis is the alteration of the cell energy metabolism. metformin inhibits mitochondrial complex I, resulting a drop in ATP production. the inhibition of ATP stimulates the activation of 5’-AMP-activated protein kinase (AMPK) [51,52,53]. All procedures applied to animals were performed in accordance with the guidelines of the Laboratory Animal Center of Ocean University of China and were approved by Animal Ethics Committee of School of Medicine and Pharmacy, Ocean University of China (Qingdao, China).

### 4.4. Glucose Tolerance Test and Insulin Tolerance Test

For the glucose tolerance test (GTT), mice were fasted for 16 h and administered orally with glucose (2 g/kg). Blood glucose was collected before and at 15, 30, 60 and 120 min. For insulin tolerance test (ITT), mice were injected with insulin (2 units/kg) after 4 h fasting. Blood glucose was collected before and at 15, 30, 45, and 60 min. Blood glucose was measured with a glucometer (Roche, Penzberg, Germany).

### 4.5. Serum Insulin Level Assay

For serum insulin level, blood was collected from mice in fed state. The concentration of serum insulin was measured by Invitrogen insulin mouse ELISA kit (Thermo Fisher, Shanghai, China).

### 4.6. Serum Biochemical Analysis

Serum samples were obtained from blood after 30 min centrifugation at 4 °C. The levels of Alanine aminotransferase (ALT) and aspartate aminotransferase (AST) were assessed by ALT and AST assay kits (Nanjing Jiancheng, Nanjing, China).

### 4.7. Western Blot analysis

Proteins from the liver tissues were measured by BCA Protein Assay Kit (Thermo Fisher, Shanghai, China) and separated by SDS-PAGE. After separated, proteins were transferred to PVDF membranes and then membranes were blocked in TBST containing 5% BSA and incubated with indicated antibodies overnight at 4 °C, followed by secondary antibody for 1 hour at room temperature. Finally, the protein bands were detected by using ECL (Nanjing Jiancheng, Nanjing, China) and were quantified by Image J.

### 4.8. Quantitative Real-time PCR

Total RNA was extracted from the liver tissues by using TRIzol reagent (Invitrogen, Carlsbad, USA) and was reversed to cDNA by using iScript cDNA Synthesis Kit (Bio-Rad, USA). Gene expression was measured with SYBR Green Supermix (Bio-Rad, Hercules, California, USA) in Bio-Rad CFX384 system. The mRNA expression was calculated by ΔΔCT methods and Cyclophilin was used as a load control. The primers used for this study were as follows, G6Pase (forward-5’- TGG TAG CCC TGT CTT TCT TTG-3’; reverse-5’- TTC CAG CAT TCA CAC TTT CCT-3’), PEPCK (forward-5’- ACA CAC ACA CAT GCT CAC AC-3’; reverse-5’- ATC ACC GCA TAG TCT CTG AA-3’), TNF-α (forward-5’-CCC GAG TGA CAA GCC TGT AG-3’; reverse-5’-GAT GGC AGA GAG GAG GTT GAC-3’), IL-6 (forward-5’-ACA GCC ACT CAC CTC TTC AG -3’; reverse-5’-CCA TCT TTT TCA GCC ATC TTT-3’), IL-1β (forward-5’-AGA TGA TAA GCC CAC TCT ACA G-3’; reverse-5’-ACA TTC AGC ACA GGA CTC TC-3’),Cyclophilin (forward-5’- AGC TAG ACT TGA AGG GGA ATG-3’; reverse-5’- ATT TCT TTT GAC TTG CGG GC-3’).

### 4.9. Statistical Analysis

All results were expressed as the mean ± SD. Statistical significance between the two groups was calculated using unpaired two-tailed t test. In Figure 2B, ANOVA tests were used to compare two groups among multiple groups. The value of *p* < 0.05 was considered statistically significantly. Data were considered no statistically significant at NS.

## References

[1] Forbes J.M., Cooper M.E. (2013). Mechanisms of diabetic complications. Physiol. Rev..

[2] Petersmann A., Nauck M., Muller-Wieland D., Kerner W., Muller U.A., Landgraf R., Freckmann G., Heinemann L. (2018). Definition, Classification and Diagnosis of Diabetes Mellitus. Exp. Clin. Endocrinol. Diabetes Off. J. Ger. Soc. Endocrinol. Ger. Diabetes Assoc..

[3] Kahn B.B., Flier J.S. (2000). Obesity and insulin resistance. J. Clin. Invest..

[4] Czech M.P. (2017). Insulin action and resistance in obesity and type 2 diabetes. Nat. Med..

[5] Adeva-Andany M.M., Perez-Felpete N., Fernandez-Fernandez C., Donapetry-Garcia C., Pazos-Garcia C. (2016). Liver glucose metabolism in humans. Biosci Rep..

[6] Biddinger S.B., Kahn C.R. (2006). From mice to men: Insights into the insulin resistance syndromes. Annu. Rev. Physiol..

[7] Rines A.K., Sharabi K., Tavares C.D., Puigserver P. (2016). Targeting hepatic glucose metabolism in the treatment of type 2 diabetes. Nat. Rev. Drug Discov..

[8] Lauritano C., Ianora A. (2016). Marine Organisms with Anti-Diabetes Properties. Mar. Drugs.

[9] Khotimchenko Y. (2018). Pharmacological Potential of Sea Cucumbers. Int. J. Mol. Sci..

[10] Janakiram N.B., Mohammed A., Rao C.V. (2015). Sea Cucumbers Metabolites as Potent Anti-Cancer Agents. Mar. Drugs.

[11] Mourao P.A., Pereira M.S., Pavao M.S., Mulloy B., Tollefsen D.M., Mowinckel M.C., Abildgaard U. (1996). Structure and anticoagulant activity of a fucosylated chondroitin sulfate from echinoderm. Sulfated fucose branches on the polysaccharide account for its high anticoagulant action. J. Biol. Chem..

[12] Wang Z., Zhang H., Yuan W., Gong W., Tang H., Liu B., Krohn K., Li L., Yi Y., Zhang W. (2012). Antifungal nortriterpene and triterpene glycosides from the sea cucumber Apostichopus japonicus Selenka. Food Chem..

[13] Zhao Y., Li B., Liu Z., Dong S., Zhao X., Zeng M. (2007). Antihypertensive effect and purification of an ACE inhibitory peptide from sea cucumber gelatin hydrolysate. Process. Biochem..

[14] Mamelona J., Pelletier É., Girard-Lalancette K., Legault J., Karboune S., Kermasha S. (2007). Quantification of phenolic contents and antioxidant capacity of Atlantic sea cucumber, Cucumaria frondosa. Food Chem..

[15] Herencia F., Ubeda A., Ferrandiz M.L., Terencio M.C., Alcaraz M.J., Garcia-Carrascosa M., Capaccioni R., Paya M. (1998). Anti-inflammatory activity in mice of extracts from Mediterranean marine invertebrates. Life Sci..

[16] Hu S., Chang Y., Wang J., Xue C., Shi D., Xu H., Wang Y. (2013). Fucosylated chondroitin sulfate from Acaudina molpadioides improves hyperglycemia via activation of PKB/GLUT4 signaling in skeletal muscle of insulin resistant mice. Food Funct..

[17] Farshadpour F., Gharibi S., Taherzadeh M., Amirinejad R., Taherkhani R., Habibian A., Zandi K. (2014). Antiviral activity of Holothuria sp. a sea cucumber against herpes simplex virus type 1 (HSV-1). Eur. Rev. Med. Pharmacol. Sci..

[18] Bordbar S., Anwar F., Saari N. (2011). High-value components and bioactives from sea cucumbers for functional foods—A review. Mar. Drugs.

[19] Mondol M.A.M., Shin H.J., Rahman M.A., Islam M.T. (2017). Sea Cucumber Glycosides: Chemical Structures, Producing Species and Important Biological Properties. Mar. Drugs.

[20] Pomin V.H. (2014). Holothurian fucosylated chondroitin sulfate. Mar. Drugs.

[21] Valcarcel J., Novoa-Carballal R., Perez-Martin R.I., Reis R.L., Vazquez J.A. (2017). Glycosaminoglycans from marine sources as therapeutic agents. Biotechnol. Adv..

[22] Yamana Y., Hamano T., Goshima S. (2009). Seasonal distribution pattern of adult sea cucumber Apostichopus japonicus (Stichopodidae) in Yoshimi Bay, western Yamaguchi Prefecture, Japan. Fish. Sci..

[23] Yang J., Wang Y., Jiang T., Lv Z. (2015). Novel branch patterns and anticoagulant activity of glycosaminoglycan from sea cucumber Apostichopus japonicus. International Journal of Biological Macromolecules.

[24] Chen Y., Liu H., Wang Y., Yang S., Yu M., Jiang T.-F., Lv Z. (2019). Glycosaminoglycan from Apostichopus japonicus inhibits hepatic glucose production via activating Akt/FoxO1 and inhibiting PKA/CREB signaling pathways in insulin resistance hepatocytes. Food Funct..

[25] Matsui Y., Hirasawa Y., Sugiura T., Toyoshi T., Kyuki K., Ito M. (2010). Metformin Reduces Body Weight Gain and Improves Glucose Intolerance in High-Fat Diet-Fed C57BL/6J Mice. Biol. Pharm. Bull..

[26] Kim Y.D., Park K.G., Lee Y.S., Park Y.Y., Kim D.K., Nedumaran B., Jang W.G., Cho W.J., Ha J., Lee I.K. (2008). Metformin inhibits hepatic gluconeogenesis through AMP-activated protein kinase-dependent regulation of the orphan nuclear receptor SHP. Diabetes.

[27] Xu H., Zhou Y., Liu Y., Ping J., Shou Q., Chen F., Ruo R. (2016). Metformin improves hepatic IRS2/PI3K/Akt signaling in insulin-resistant rats of NASH and cirrhosis. J. Endocrinol..

[28] Zhao Y., Hu X., Liu Y., Dong S., Wen Z., He W., Zhang S., Huang Q., Shi M. (2017). ROS signaling under metabolic stress: Cross-talk between AMPK and AKT pathway. Mol. Cancer.

[29] Hu S.W., Tian Y.Y., Chang Y.G., Li Z.J., Xue C.H., Wang Y.M. (2014). Fucosylated chondroitin sulfate from sea cucumber improves glucose metabolism and activates insulin signaling in the liver of insulin-resistant mice. J. Med. Food.

[30] Hu S., Chang Y., Wang J., Xue C., Li Z., Wang Y. (2013). Fucosylated chondroitin sulfate from sea cucumber in combination with rosiglitazone improved glucose metabolism in the liver of the insulin-resistant mice. Biosci. Biotechnol. Biochem..

[31] Zhu Z., Zhu B., Sun Y., Ai C., Wang L., Wen C., Yang J., Song S., Liu X. (2018). Sulfated Polysaccharide from Sea Cucumber and its Depolymerized Derivative Prevent Obesity in Association with Modification of Gut Microbiota in High-Fat Diet-Fed Mice. Mol. Nutr. Food Res..

[32] Han H.S., Kang G., Kim J.S., Choi B.H., Koo S.H. (2016). Regulation of glucose metabolism from a liver-centric perspective. Exp. Mol. Med..

[33] Petersen M.C., Vatner D.F., Shulman G.I. (2017). Regulation of hepatic glucose metabolism in health and disease. Nat. Rev. Endocrinol..

[34] Meshkani R., Adeli K. (2009). Hepatic insulin resistance, metabolic syndrome and cardiovascular disease. Clin. Biochem..

[35] Lin H.V., Accili D. (2011). Hormonal regulation of hepatic glucose production in health and disease. Cell. Metab..

[36] Manning B.D., Toker A. (2017). AKT/PKB Signaling: Navigating the Network. Cell.

[37] Dong X.C., Copps K.D., Guo S., Li Y., Kollipara R., DePinho R.A., White M.F. (2008). Inactivation of hepatic Foxo1 by insulin signaling is required for adaptive nutrient homeostasis and endocrine growth regulation. Cell. Metab..

[38] Lin S.C., Hardie D.G. (2018). AMPK: Sensing Glucose as well as Cellular Energy Status. Cell. Metab..

[39] Collins Q.F., Liu H.Y., Pi J., Liu Z., Quon M.J., Cao W. (2007). Epigallocatechin-3-gallate (EGCG), a green tea polyphenol, suppresses hepatic gluconeogenesis through 5’-AMP-activated protein kinase. J. Biol. Chem..

[40] Kim S.J., Yuan H.D., Chung S.H. (2010). Ginsenoside Rg1 suppresses hepatic glucose production via AMP-activated protein kinase in HepG2 cells. Biol. Pharm. Bull..

[41] Vlavcheski F., Naimi M., Murphy B., Hudlicky T., Tsiani E. (2017). Rosmarinic Acid, a Rosemary Extract Polyphenol, Increases Skeletal Muscle Cell Glucose Uptake and Activates AMPK. Molecules.

[42] Zhang R., Qin X., Zhang T., Li Q., Zhang J., Zhao J. (2018). Astragalus Polysaccharide Improves Insulin Sensitivity via AMPK Activation in 3T3-L1 Adipocytes. Molecules.

[43] Shoelson S.E., Lee J., Goldfine A.B. (2006). Inflammation and insulin resistance. J. Clin. Invest..

[44] Cai D., Yuan M., Frantz D.F., Melendez P.A., Hansen L., Lee J., Shoelson S.E. (2005). Local and systemic insulin resistance resulting from hepatic activation of IKK-beta and NF-kappaB. Nat. Med..

[45] Hirosumi J., Tuncman G., Chang L., Gorgun C.Z., Uysal K.T., Maeda K., Karin M., Hotamisligil G.S. (2002). A central role for JNK in obesity and insulin resistance. Nature.

[46] Gao Z., Zuberi A., Quon M.J., Dong Z., Ye J. (2003). Aspirin inhibits serine phosphorylation of insulin receptor substrate 1 in tumor necrosis factor-treated cells through targeting multiple serine kinases. J. Biol. Chem..

[47] Nakamura T., Furuhashi M., Li P., Cao H., Tuncman G., Sonenberg N., Gorgun C.Z., Hotamisligil G.S. (2010). Double-stranded RNA-dependent protein kinase links pathogen sensing with stress and metabolic homeostasis. Cell.

[48] Yuan M., Konstantopoulos N., Lee J., Hansen L., Li Z.W., Karin M., Shoelson S.E. (2001). Reversal of obesity- and diet-induced insulin resistance with salicylates or targeted disruption of Ikkbeta. Sci. (New York, N.Y.).

[49] Imanari T., Washio Y., Huang Y., Toyoda H., Suzuki A., Toida T. (1999). Oral absorption and clearance of partially depolymerized fucosyl chondroitin sulfate from sea cucumber. Thromb. Res..

[50] Pernicova I., Korbonits M. (2014). Metformin--mode of action and clinical implications for diabetes and cancer. Nat. Rev. Endocrinol..

[51] El-Mir M.Y., Nogueira V., Fontaine E., Avéret N., Rigoulet M., Leverve X. (2000). Dimethylbiguanide inhibits cell respiration via an indirect effect targeted on the respiratory chain complex I. J. Biol. Chem..

[52] Miller R.A., Birnbaum M.J. (2010). An energetic tale of AMPK-independent effects of metformin. J. Clin. Investig..

[53] Rena G., Hardie D.G., Pearson E.R. (2017). The mechanisms of action of metformin. Diabetologia.

